# Dual Utilization of Lignocellulose Biomass and Glycerol Waste to Produce Fermentable Levoglucosan via Fast Pyrolysis

**DOI:** 10.3389/fchem.2022.847767

**Published:** 2022-03-10

**Authors:** Yingchuan Zhang, Feixiang Xu, Fenglin Chen, Yanru Zhang, Yaxiang Wu, Liqun Jiang

**Affiliations:** ^1^ State Key Laboratory of Utilization of Woody Oil Resource, Hunan Academy of Forestry, Changsha, China; ^2^ Institute of Biological and Medical Engineering, Guangdong Academy of Sciences, Guangzhou, China; ^3^ Guangzhou Institute of Energy Conversion, Chinese Academy of Sciences, Guangzhou, China; ^4^ Department of Chemistry, The University of Hong Kong, Hong Kong SAR, China; ^5^ College of Biology, Hunan University, Changsha, China

**Keywords:** lignocellulose biomass, glycerol waste, microwave pretreatment, levoglucosan, fast pyrolysis

## Abstract

Glycerol waste was combined with microwave to pretreat lignocellulose before fast pyrolysis. After pretreatment, most alkali and alkaline earth metals (87.9%) and lignin (52.6%) were removed, and a higher crystallinity was obtained. Comparatively, glycerol waste combined with microwave was proven to be more efficient than glycerol with conventional heating. During fast pyrolysis, higher content of levoglucosan in glycerol waste–pretreated products (27.5%) was obtained, compared with those pretreated by pure glycerol (18.8%) and untreated samples (5.8%). Production of fermentative toxic aldehyde and phenol by-products was also inhibited after glycerol waste treatment. Following mechanistic study had validated that microwave in glycerol waste solvent could effectively ameliorate structure and components of lignocellulose while selectively removing lignin. Notably, under the optimal condition, the levoglucosan content in pyrolytic products was enhanced significantly from 5.8% to 32.9%. In short, this study provided an archetype to dually utilize waste resources for ameliorating lignocellulose structure and precisely manipulating complex fast pyrolysis.

## Introduction

Because of the global energy and environmental issues, development of environment-friendly technology for utilizing sustainable resources is urgently needed (Shen et al., 2019; Qian et al., 2021). Lignocellulose, as a predominant natural carbon source with a worldwide distribution, can potentially provide versatile carbohydrate resources. A traditional way of valorizing lignocellulose is to hydrolyze it to fermentable sugars by enzymes or acids and then convert those into valued chemicals via large-scale fermentation (Clomburg et al., 2017), whereas technical limitations, such as a tedious and expensive process and a low yield of fermentable products versus uncontrollable by-products, restrict the viability of industrial intermediation of lignocellulose (Lin and Lu, 2021).

Emerging in recent years, pyrolysis harnesses the thermochemical power to unleash numerous energy-hindered chemical reactions. As a widely used pyrolysis technology, fast pyrolysis is typically performed under temperature of 500°C–750°C with a rapid heating rate (100°C/s–500°C/s) and a short residence time (<2 s), which greatly expand its applications for a quick and efficient thermal process. For its utilization in lignocellulose conversion, it has been reported that fast pyrolysis of cellulose can yield up to 70.1 wt%-concentrated commercial levoglucosan for further fermentation (Kwon et al., 2007). As an intriguing anhydride form of glucose, levoglucosan can be easily fermented to a variety of chemicals by both prokaryotic and eukaryotic enzymes or bioreactors and next fermented to ethanol, lipids, and itaconic acid (Jabareen et al., 2021). The economics of fast pyrolysis technology to obtain fermentable intermediates from biomass and further conversion has been evaluated to be competent with traditional direct enzymatic and acid hydrolysis (Vivek et al., 2017).

To further enhance the performance of lignocellulose-derived intermediates for fermentation, an efficient production of levoglucosan is of great significance during fast pyrolysis. Levoglucosan can be formed by cleavage of β-1,4-glycosidic bonds in cellulose under homolytic, heterolytic, or concerted mechanism (Jiang et al., 2017). Unfortunately, release of this anhydride sugar via fast pyrolysis is hindered by the intrinsic obstructive structure of lignocellulose in biomass. A reality is that production of levoglucosan from lignocellulose is far more difficult than pure crystalline cellulose. Many studies have shown that both metal ashes and lignin components in natural lignocellulose could impede the thermo-induced destabilization of obstructive structure and promote the fast pyrolysis of lignocellulose to generate small molecule compounds such as ketones and aldehydes instead of levoglucosan (Zhang et al., 2015; Maduskar et al., 2018). Besides, extra separation and purification for removing these by-products are necessary before fermentation, as C1-C3 aldehydes have been proven with capacity of inhibiting hydrolytic enzymes and killing microorganisms (Zaldivar et al., 1999; Xie et al., 2016). To solve this problem, various pretreatment methods have been developed to ameliorate the component and structure of lignocellulose for the following selective pyrolysis. In general, they can be classified as physical methods (by grinding or high-energy radiation), chemical methods (via acid/alkali or oxidative treatment), and hybrid methods (such as steam explosion hydrothermal and organosolv treatment) (Jiang et al., 2019). Among all hybrid methods are notably highlighted because of its simple and flexible process, excellent and selective amelioration, and cheap and sustainable possibility. A key problem, however, is the choice of chemical solvent to bridge the physical process, where a large range from acid–base to ionic liquids have been explored and indeed achieved different valorization effects.

Disposal of glycerol waste is a long-term perplexing environmental issue, with toilsome degradation contrary to its wide use in medicine, cosmetics, and other fields (Anitha et al., 2016). Compositionally, 70% to 98% in glycerol waste is glycerin, along with low extent of fatty acids, methyl esters, fatty acids, alcohols, and inorganic salts. Its high boiling point and hydrophobicity consequently hinder a thorough decomposition and degradation. Recently, a novel reutilization method using glycerol to pretreat lignocellulose under atmospheric pressure followed by fast pyrolysis has been developed, where selective and enhanced production of levoglucosan has been achieved (Jiang et al., 2017; Jiang et al., 2020). Glycerol provided an excellent thermal medium when pretreating lignocellulose; external heating is necessary for ameliorating its obstacle structure. Recently, it has also been reported that a biomass hydrolysis approach that dilutes acid-catalyzed glycerol pretreatment can significantly enhance the production of microbial oils by removing lignin-degradation products (Hassanpour et al., 2020).

**FIGURE 1:**
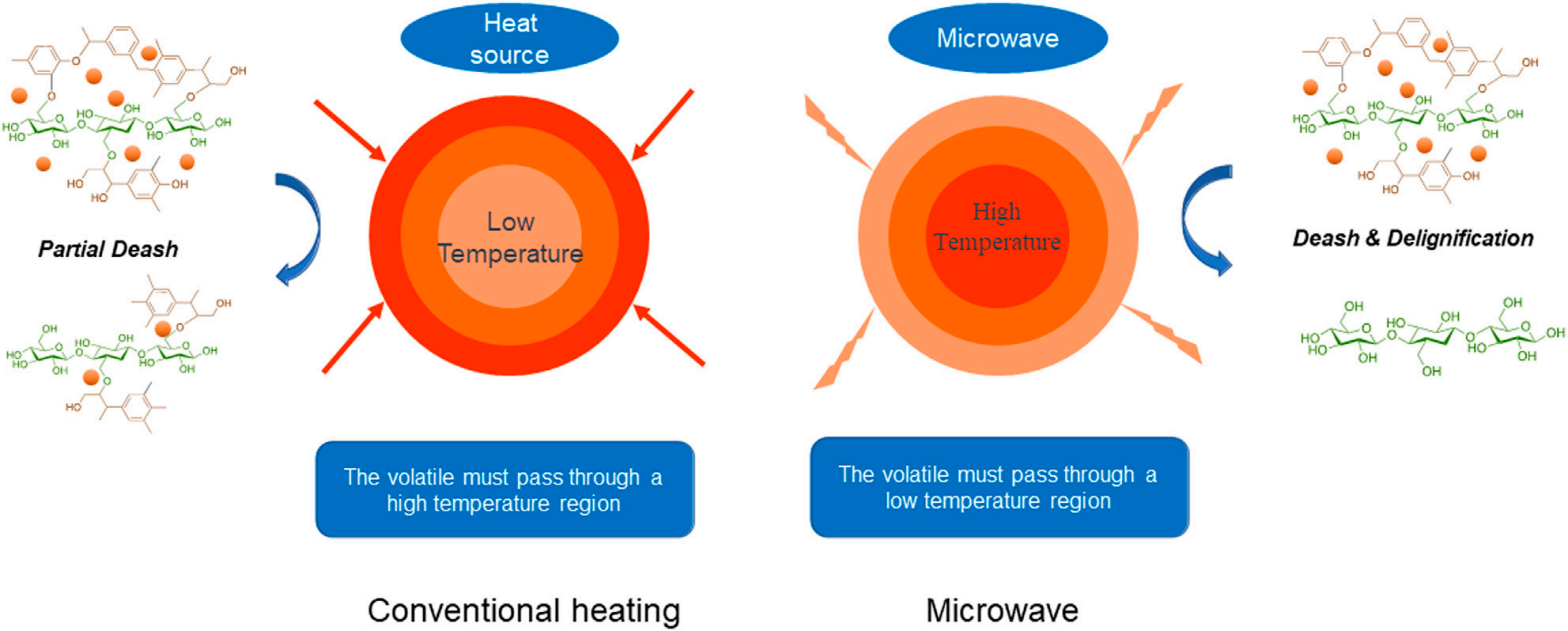
Schematic illustration of conventional heating and microwave treatments.

With regard to thermal conversion compared with traditional methods, mild microwave can effectively destroy the recalcitrant structure of lignocellulose due to volumetric heating and selective penetration (Hu and Wen, 2008). In microwave process, high-frequency reciprocation of dipole molecules can go inside the heated materials to generate the internal frictional heat that quickly raises the intrinsic temperature (Figure 1). Unfortunately, dried lignocellulose is not prone to microwave energy due to the poor dielectric properties and shakable components. It has been illustrated that cotreatment with glycerol with a high boiling point and hydrophobicity can handle the dielectric nature of lignocellulose, thus improving the pretreatment effects (Zhou et al., 2017).

Herein, obstinate lignocellulose (reed rod) was soaked with glycerol waste and then cotreated under different conditions for amelioration before fast pyrolysis. The consequent structural and componential changes and following fast pyrolytic reactions were extensively investigated. Production of levoglucosan and small molecule compounds with capacity to inhibit fermentation was systematically quantified and compared. Mechanism behind these effects was revealed via elemental, componential, and crystallinity analysis. These studies provided an insight into the precise manipulation of fast pyrolysis of lignocellulose via glycerol waste and microwave pretreatment.

## Materials and Methods

### Materials

Reed rod was collected from Hunan province, China. Before use, it was pulverized to 60–80 mesh and then dried at 105°C to obtain constant weight. Glycerol was obtained from Tianjin Yongda Chemical Reagent Co., Ltd. Other agents were purchased from Sigma–Aldrich (Shanghai). Glycerol waste was prepared in laboratory by transesterification of soybean oil and methanol with the catalyst of sodium silicate (Na_2_SiO_3_·9H_2_O). Methanol was mixed with soybean oil and catalyst at mole ratios of 15:1 and 40:1, respectively. The reaction was performed at 65°C for 3 h. The components of glycerol waste had been reported in previous literature (Jiang et al., 2017).

### Pretreatment Procedure

Microwave treatment was executed in microwave reactor (MCR-3, Guangzhou Xingshuo Instrument Co., Ltd., China). Reed rod and glycerol were added to a 250-mL flat-bottomed double-necked flask at a solid–liquid ratio of 1:20 (wt/wt) and well-mixed with a glass rod. To prevent the volatilization of glycerol, a reflux device was connected. PW-220 and PW-160 represented microwave treatment with glycerol at 220°C and 160°C for 6 min, whereas BW-160 referred to microwave treatment with glycerol waste at 160°C for 6 min. Heating treatment was performed in an oil bath. Similarly, glycerol and reed rod were mixed thoroughly before pretreatment. PY-160 and BY-160 represented oil bath treatment with glycerol and glycerol waste heated at 160°C for 6 min, respectively.

After pretreatment, the residual glycerol and soluble substances in the reed rod sample were thoroughly washed with deionized water. The solid residue was collected by vacuum filtration and then dried with a vacuum freeze dryer. The recovery rate of pretreated solid samples was evaluated as follows:

$$\text{Recovery rate}\left(\text{wt\%}\right)=\frac{\text{solid mass after treatment }}{\text{solid mass before treatment}}\times 100\%$$ (1)

### Elemental and Componential Analysis

Elemental analyzer (Vario EL cube; Elementar, Germany) was used to determine the content of the main organic elements (C, H, N) in lignocellulose. Content of alkali and alkaline earth metals (AAEMs) was detected by an inductively coupled plasma emission spectrometer (OPTIMA 8000DV; PerkinElmer, United States). Components of cellulose and hemicellulose were quantified according to the technical report issued by the National Renewable Energy Laboratory using Sugar Column (Aminex HPX-87P) using formulas (2) and (3) (Sluiter et al., 2004). Lignin content was measured by detecting the absorbance of the liquid phase portion under λ = 320 nm and weighing the insoluble ash after burning. The content of lignin was defined as the sum of acid-soluble and acid-insoluble lignin parts. To demonstrate the removing effect of pretreatment, a component removal rate was introduced, and its definition is shown in formula (4)

$$\text{Cellulose content }\left(\text{wt\%}\right)=\frac{\text{Glucose conc}.\;\left(\text{g}/\text{L}\right)\times 0.90\times \text{liquid volume }\left(\text{L}\right)\;}{\text{Biomass total mass }\left(\text{g}\right)}\times 100\%$$ (2)

$$\text{Hemiellulose content }\left(\text{wt\%}\right)=\frac{\text{Xylose conc}.\;\left(\text{g}/\text{L}\right)\times 0.88\times \text{liquid volume }\left(\text{L}\right)\;}{\text{Biomass total mass }\left(\text{g}\right)}\times 100\%$$ (3)

$$\text{Removal rate }\left(\text{\%}\right)=\frac{m_{1}C_{1}-m_{2}C_{2}}{m_{1}C_{1}}\times 100\%$$ (4)

where *m*_1_ and *m*_2_ are the sample mass before and after hydrolysis, respectively. Similarly, *C*_1_ and *C*_2_ refer to the content of specific component before and after hydrolysis, respectively.

### Crystallinity Analysis

The crystallinity of samples was analyzed by X-ray diffractometer (PANalytical V.B., Holland) with Cu radiation (λ = 1.54Å). The tube voltage and current of the diffractometers were 40 kV and 40 mA. The scanned range of the 2θ angle for X-ray was 5° to 45°. The crystallinity was calculated according to formula (5).

$$\text{CrI }\left(\text{\%}\right)=\frac{I_{002}-I_{\text{am}}}{I_{002}}\times 100\%$$ (5)

where CrI and *I*_002_ represent the crystallinity index, *I*_002_ refers to the peak intensity (2θ = 22°) representative for crystalline and amorphous cellulose, and *I*_am_ refers to the peak intensity (2θ = 18°) for amorphous cellulose.

### Fast Pyrolysis

Fast pyrolysis was performed with semi-batch CDS reactor (Pyroprobe 5200; CDS Analytical, United States) in duplicate. Reaction temperature was set at 500°C, and heating rate was 20 K · ms^−1^. High-purity helium was set at 20 mL/min to sweep the fast pyrolyzed volatiles through a 300°C transmission line into the gas chromatography–mass spectrometry (GC-MS) system (GC-7890A, MS-5975C; Agilent Technologies, United States) for separation. Specifically, pyrolysis volatiles were separated by DB-1701 capillary column. GC oven temperature was set to be maintained at 40°C for 3 min and then increased from 40°C to 280°C at a rate of 5°C/min and finally at 280°C for 8 min. Mass spectrometer parameters include ion source temperature of 230°C, quadrupole temperature of 150°C, ion source energy of 70 eV, and a scan range (m/z) of 29 to 450 amu. The relative content of compounds was calculated according to formula (6).

$$\text{Relative content }\left(\text{\%}\right)=\frac{\text{Area of specific compound }}{\text{Total area of all compounds}}\times 100\%$$ (6)

## Results and Discussion

### Elemental Analysis

Content of three dominant organic elements in lignocellulose is shown in Table 1. In untreated sample, contents of C (46.4 wt%), H (5.8 wt%), and N (0.1 wt%) were detected, where C/H ratio was calculated as 8.0. In pretreated samples, C (45.3–46.7 wt%), H (6.1–6.3 wt%), and N (0.0 wt%) were given, consequently with a dropped C/H ratio to 7.2 to 7.7. It had been reported that C/H ratio in lignin was much higher than those in cellulose and hemicellulose (11.1 compared with 7.5 and 7.2) (Maduskar et al., 2018). Furthermore, microwave and glycerol waste condition was found to be more effective in reducing the organic element contents and C/H ratio than heating and pure glycerol (7.7 vs. 7.3). This discrimination was inferred with relation to the polar nature of glycerol waste to preferably absorb AAEMs ions, derived from the multicomponent of carboxylic acids and metals.

**TABLE 1:** Organic elemental analysis of different sample.

Sample	C (wt%)	H (wt%)	N (wt%)	C/H
Untreated	46.4	5.8	0.1	8.0
PW-220	45.3	6.3	ND	7.2
PW-160	46.0	6.1	ND	7.5
PY -160	46.7	6.1	ND	7.7
BW-160	45.8	6.3	ND	7.3
BY -160	46.1	6.1	ND	7.6

ND, means not detected.

As for AAEMs, it has been reported that these metals were contained massively in natural biomass and had a significant catalytic effect on the pyrolytic reaction that consequently influenced the product distribution. Content of AAEMs is shown in Table 2. In untreated biomass, contents of K, Ca, Na, and Mg were 3,396.2, 1,055.8, 543.2, and 231.2 mg/kg (kg is the unit of biomass), respectively, and the overall value was 5,226.4 mg/kg. After glycerol treatment, the overall value was decreased to 1,154.9 to 1,903.2 mg/kg with a removal rate of 63.6% to 77.9%. Comparatively, in glycerol waste–pretreated samples, it was decreased more effectively, with 635.8 to 702.0 mg/kg with removal by 86.6% to 87.8%. Furthermore, as the heating temperature was lifted to 220°C, an optimal removal of AAEMs was found with an absolute removal of K up to 97.3% to 99.4%.

**TABLE 2:** Inorganic elemental analysis of different samples.

Samples	AAEMs (mg/kg)					Removal rate (%)
	K	Ca	Na	Mg	Total	
Untreated	3,396.2	1,055.8	543.2	231.2	5,226.4	—
PW-220	70.8	656.6	353.5	74.0	1,154.9	77.9
PW-160	59.6	976.0	424.2	143.6	1,603.4	69.3
PY-160	20.7	1,309.7	386.2	186.6	1,903.2	63.6
BW-160	91.0	230.1	353.9	27.0	702.0	86.6
BY-160	66.2	222.2	322.1	25.3	635.8	87.8

Content of AAEMs had a huge impact on the yield of levoglucosan, as trace existence of AAEMs could inhibit the pathway to form levoglucosan while promoting its competitive reaction for the generation of small molecular compounds (Kuzhiyil et al., 2012; Lindstrom et al., 2019). In detail, the levoglucosan formation depended on cleavage of bonds between cellulose units, but during the competitive reaction, the pyranose rings in each monomer were intrinsically opened (Patwardhan et al., 2010). It has been found that AAEMs potentially generate coordinate complex with hydroxyl groups located at the ortho-positions in pyranose rings, leading to its structural stabilization and conversely antagonizing the production of levoglucosan (Shaik et al., 2013). Among AAEMs, K was reported to be the most effective factor to inhibit production of anhydride sugars (Patwardhan et al., 2010). In glycerol waste–treated samples, it was efficiently removed compared with other AAEMs, which confirmed the fundamental for the power of glycerol pretreatment to enhance levoglucosan production.

### Componential Analysis

**FIGURE 2:**
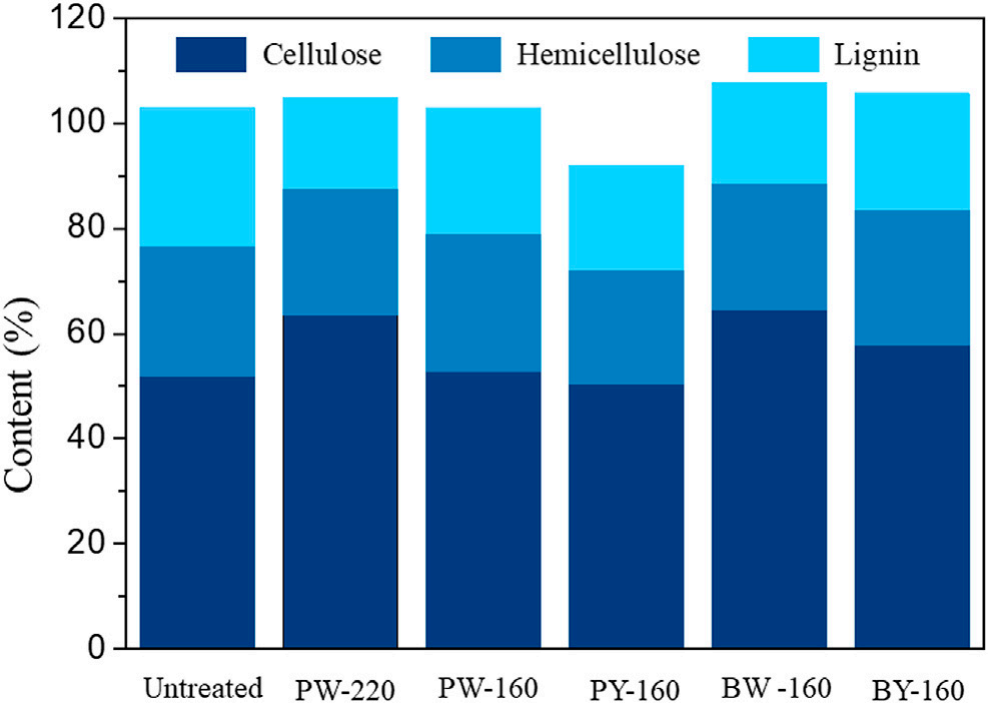
Content of major components after different pretreatment.

**FIGURE 3:**
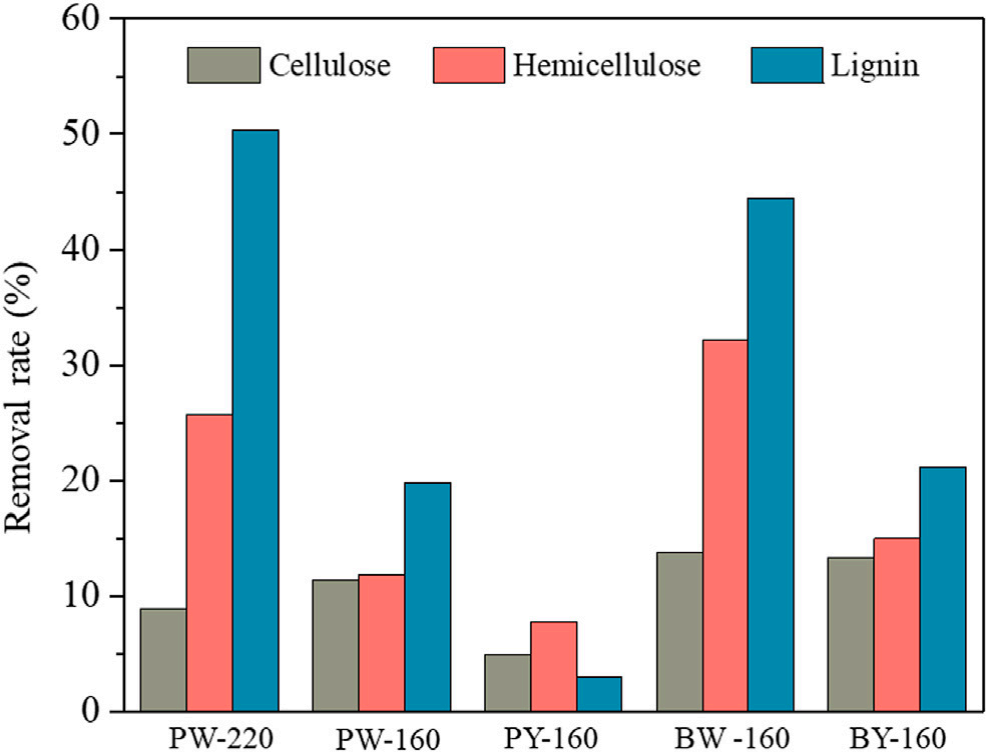
Removal rates of major components after different pretreatment.

Calculated recovery rates of pretreated biomass variously were from 72.9 to 99.0 wt%, which validated the analytical confidence. Distribution of three cellulose, hemicellulose, and lignin in different samples is demonstrated in Figure 2. Despite an overall removal effect of three components after pretreatment, the most significantly removed component was lignin, which dropped from 26.4% in natural biomass to 17.1% to 23.8% in pretreated samples and achieved the lowest content in PW-220 sample treated with glycerol waste and microwave. To intuitively compare the removal effect of different pretreatment, componential proportions with recovery rates are shown in Figure 3. For PW-220, cellulose and hemicellulose were removed only by 2.0% to 10.5% and 4.6% to 29.5% compared with lignin by 52.6%. After comparing the removal rates in samples treated at 160°C, it was found that the removal of lignin was more efficient in glycerol waste–treated samples than those with pure glycerol treatment. This was probably due to the complex component in glycerol waste, which could facilitate the dissolution of hydrophobic aromatic lignin. According to previous research, glycerol waste derived from biodiesel had a peculiar composition of (Z, Z)-9,12-octadecadienoic acid, n-hexadecenoic acid, and 4-methyl-2-pentanol, which are intermiscible with lignin and also alkaline metals (Wu et al., 2019). In addition, higher temperature was found to favor the delignification in lignocellulose. These results were also consistent with C/H ratio change revealed in previous elemental analysis. It has been reported that removal of lignin could significantly increase the yield of levoglucosan due to the enhanced relative content of cellulose (Miura et al., 2001). Simultaneously, chemical linkages between cellulose–hemicellulose and cellulose–lignin had a profound impact on the distribution of pyrolytic products in varying degrees. These covalent bonds between cellulose and lignin would significantly inhibit the production of levoglucosan, instead of promoting the generation of small molecule compounds (Hosoya et al., 2007).

### Crystallinity Analysis

**FIGURE 4:**
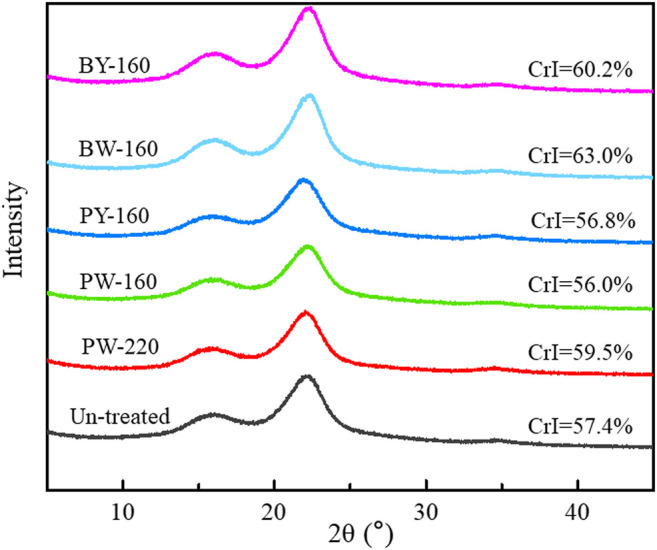
X-ray diffraction spectra of lignocellulose samples after different pretreatment.

X-ray diffraction spectra of lignocellulose before and after glycerol treatment are shown in Figure 4. Effects on crystallinity were reported to correlate with both the components and structure of raw material, as well as the treatment conditions including times, temperatures, and ratios of aqueous/solid segments (Jiang et al., 2019). As a referable index of pyrolysate distribution, the crystallinity of biomass was increased from 57.4% to 63.0% after glycerol treatment. The maximum peak was achieved in BW-160 that was treated by glycerol waste with microwave. Notably, an efficient removal of lignin and hemicellulose in this sample aforementioned was consistent with its high crystallinity (Maduskar et al., 2018). In brief, enhancement on crystallinity by microwave was found to be more effective than oil bath heating, and higher temperature favored this effect.

The improvement of crystallinity was attributed to the selective removal of amorphous lignin and hemicellulose segments, simultaneously conserving the crystalline cellulose by glycerol waste with hydrophobic interactions. As levoglucosan was sourced from the cellulose, a conserved crystalline structure could significantly profit its release. As reported, cellulose type I (with a higher crystallinity) tended to be transferred to levoglucosan more efficiently, whereas cellulose with a lower crystallinity favored the conversion to carbonyl compounds or 5-methyl furfural (Mukarakate et al., 2016). Consistent with previous study, microwave was remarkable in removing amorphous components, superior to traditional thermal conduction way (Shi et al., 2011). This study also mentioned that removal of amorphous components could improve the pyrolytic yield of levoglucosan, which was demonstrated in the following analysis.

### Fast Pyrolysis

**FIGURE 5:**
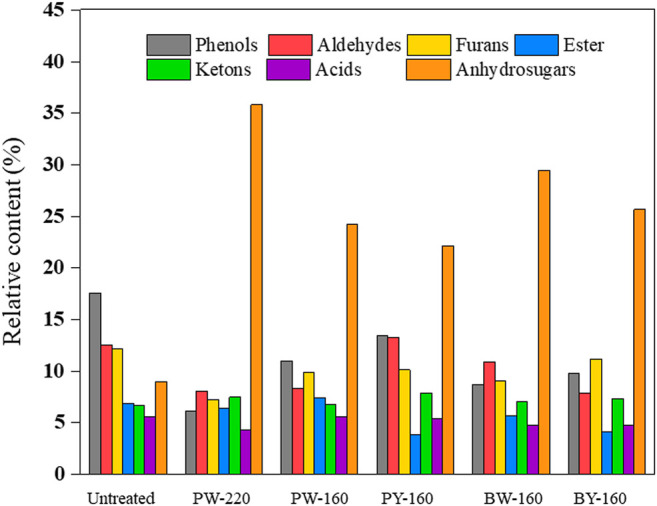
Product distribution for fast pyrolysis of different samples.

Pyrolytic products consisted of several organic compounds from C1 to C5 (listed in Table 3). Up to 100 compounds identified by GC-MS were divided into seven groups (acids, esters, furans, aldehydes, ketones, phenols, and anhydrosugars) in Figure 5. There was no apparent change in the product repertoire after different pretreatment. However, the proportion of phenols in pyrolytic products declined from 17.6% to a minimum of 6.2% in glycerol and microwave-treated samples. More detailed, phenol, 2-methoxy-4-vinyl phenol, 2,6-dimethoxy- phenol, and 4-((1E)-3-hydroxy-1-propenyl)-2-methoxy phenol exhibited a decrease of 50% compared with untreated sample. This decrease was attributed to the removal of lignin, as aromatic network was destroyed and removed by glycerol waste. The lowest proportion of phenols in pyrolytic products was achieved in PW-220, which was in accordance with the componential and elemental analysis. Production of fermentative toxic aldehydes was also inhibited, from 4.3% in untreated to minimum 2.6% after glycerol pretreatment. Besides, there was a slight decrease in the production of acids (5.6% before pretreatment and the lowest in PW-220 as 4.3% after pretreatment), especially acetic acid, which had a decrease up to 81.6%. In contrast, an increase in acids such as adenosine and N6-phenylacetic acid was observed conversely. The content of anhydrosugars accounted for 9.0% in untreated sample and 22.2% to 35.9% in pretreated samples. Notably, the production of 1,6-anhydro-β-d-glucofuranose (0.2–1.2%) was negligible as compared with levoglucosan, which constituted the major component of anhydrosugars. Bearing pyrolysis, the content of levoglucosan was only 5.8% in the untreated sample, which was disappointingly less than lignin derivative 2,3-dihydro-benzofuran with content of 8.4%. After glycerol treatment, the latter dropped to 2.8% while significantly raising the generation of levoglucosan. Its content in pyrolytic products was increased by 5.7-fold in PW-220 (32.9%). By comparing PW-220 and PW-160, it could be found that an upper temperature was crucial, with an enhancement of 11.4% in the levoglucosan yield using microwave. Looking back to the elemental and componential analysis, both an effective removal of lignin and AAEMs at high temperatures contributed to this boosted yield. Furthermore, it was found that glycerol waste was more beneficial to the production of levoglucosan. Under the same condition, the pyrolytic yield from glycerol waste–pretreated samples was 27.5% than 21.5% from pure glycerol-treated samples. A similar trend was observed in oil bath–heated samples, as 23.0% came from glycerol waste samples compared with 18.8% pretreated by pure glycerol. There was a noticeable difference in the production of levoglucosan between treatment using microwave and oil bath. After the pure/crude glycerol pretreatment, relative content of levoglucosan from samples heated by microwave was 21.5%/27.5%, compared with 18.8%/23.0% in the oil bath–heated samples. It was concluded that the removal of lignin derived from glycerol was further enhanced by microwave.

**TABLE 3:** Relative content of major pyrolytic products from different samples.

Retention time (min)	Compounds	Relative content (%)					
		Untreated	PW-220	PW-160	PY-160	BW-160	BY-160
4.8	Methyl glyoxal	1.8	3.2	3.6	3.4	3.1	3.1
7.0	Acetaldehyde, hydroxy-	4.3	3.5	2.9	2.6	5.3	3.3
8.2	Acetic acid	3.8	0.7	2.9	3.5	0.7	1.5
12.3	Acetic acid, methyl ester	1.5	0.6	1.6	1.4	0.4	0.7
14.3	Furfural	1.3	1.3	1.4	1.5	1.8	1.7
15.7	2(3H)-furanone, 5-methyl-	0.1	0.1	0.1	0.1	0.1	0.1
15.8	2-Furanmethanol	0.3	0.1	0.1	0.2	0.3	0.2
18.0	1,2-Cyclopentanedione	1.7	0.9	1.0	1.1	1.3	1.2
19.7	2(5H)-furanone	0.6	0.2	0.3	0.3	0.6	0.4
22.0	Phenol	0.8	0.2	0.3	0.4	0.3	0.3
22.5	Phenol, 2-methoxy-	1.7	0.5	0.8	0.9	0.8	0.8
24.4	*p*-Cresol	0.3	0.5	0.6	0.7	0.5	0.5
25.5	Creosol	0.6	0.4	0.6	0.6	0.7	0.5
26.9	Phenol, 4-ethyl-	0.3	0.1	0.3	0.3	0.2	0.3
28.3	2,3-Anhydro-d-mannosan	0.4	0.1	0.6	0.7	0.4	0.6
28.7	1,4:3,6-Dianhydro-α-d-glucopyranose	0.4	0.4	0.3	0.4	0.4	0.4
29.2	Benzofuran, 2,3-dihydro-	8.4	2.8	6.5	6.7	3.1	7.0
29.3	2-Methoxy-4-vinylphenol	3.7	1.8	2.8	3.1	1.9	2.6
30.3	5-Hydroxymethylfurfural	0.2	0.7	0.5	0.6	1.0	0.8
30.7	Phenol, 2,6-dimethoxy-	3.1	0.7	1.3	1.5	1.3	1.5
32.9	3,5-Dimethoxy-4-hydroxytoluene	0.8	0.5	0.8	0.8	0.8	0.8
36.5	(E)-2,6-dimethoxy-4-(prop-1-en-1-yl)-phenol	1.9	1.0	1.7	1.7	1.5	1.5
38.6	Levoglucosan	5.8	32.9	21.5	18.8	27.5	23.0
39.6	Benzaldehyde, 4-hydroxy-3,5-dimethoxy-	0.7	0.2	0.4	0.5	0.4	0.4
41.7	1,6-Anhydro-β-d-glucofuranose	0.2	1.2	0.5	0.5	0.8	0.8

Considering all effects mentioned previously, enhanced pyrolytic production of levoglucosan could be explained as (1) the catalytic effect on promoting competitive reactions against levoglucosan production was prohibited with the removal of AAEMs; (2) relative abundance of lignin was increased due to the selective removal of lignin; and (3) the thermal stability was improved with increased crystallinity of reactants (Wu et al., 2019). These effects consequently decreased the conversion of cellulose into small molecule compounds and promoted the formation of levoglucosan. Besides, reactions responsible for the productions of aldehydes were inhibited, which could favor the following fermentation process. This could maximally improve the fermentability of anhydrosugars from pyrolysis, leading to an economical downstream to further valorize biomass products.

## Conclusion

In this work, glycerol waste was comparatively used to evaluate the ameliorative effect on lignocellulose under microwave or conventional heating method, and the following fast pyrolysis was comprehensively assessed. In elemental, componential, and crystallinity analysis, lignocellulose pretreated by glycerol waste was found to be ameliorated thoroughly. Intriguingly, during fast pyrolysis, production of levoglucosan from microwave-treated samples (32.9%) was far more selective than conventional heating group (18.8%). Furthermore, content of aldehydes with high toxicity to the downstream fermentation was decreased to 2.5 times the untreated after glycerol waste and microwave pretreatment. In summary, this work provided an elaborate insight into the precise manipulation of fast pyrolysis of lignocellulose via glycerol waste and microwave treatment and could provoke more novel approaches to ameliorating lignocellulose in the future.

## Data Availability

The original contributions presented in the study are included in the article/supplementary material, further inquiries can be directed to the corresponding author.
